# Another Really, Really Big Virus

**DOI:** 10.3390/v3010032

**Published:** 2011-01-18

**Authors:** James L. Van Etten

**Affiliations:** Department of Plant Pathology, Nebraska Center for Virology, 205 Morrison Hall, University of Nebraska, Lincoln, NE 68583, USA; Email: jvanetten@unlnotes.unl.edu; Tel. +1 402 472 3168

**Keywords:** giruses, NCLDV, huge viruses

## Abstract

Viruses with genomes larger than 300 kb and up to 1.2 Mb, which encode hundreds of proteins, are being discovered and characterized with increasing frequency. Most, but not all, of these large viruses (often referred to as giruses) infect protists that live in aqueous environments. Bioinformatic analyses of metagenomes of aqueous samples indicate that large DNA viruses are quite common in nature and await discovery. One issue that is perhaps not appreciated by the virology community is that large viruses, even those classified in the same family, can differ significantly in morphology, lifestyle, and gene complement. This brief commentary, which will mention some of these unique properties, was stimulated by the characterization of the newest member of this club, virus CroV (Fischer, M.G.; Allen, M.J.; Wilson, W.H.; Suttle, C.A. Giant virus with a remarkable complement of genes infects marine zooplankton. *Proc. Natl. Acad. Sci. USA* **2010**, *107*, 19508–19513 [1]). CroV has a 730 kb genome (with ∼544 protein-encoding genes) and infects the marine microzooplankton *Cafeteria roenbergensis* producing a lytic infection.

## 1. Introduction

Typically, viruses are considered to be small particles that easily pass through 0.2 μm filters and have small genomes containing a few protein-encoding genes. However, large viruses with huge dsDNA genomes that encode hundreds of proteins are being discovered with increasing frequency. These large viruses have also been referred to as giruses in order to emphasize their unique properties [2]. Examples of giruses include: i) Mimivirus and its close relative Mamavirus, which infect amoebae and have the largest genomes (∼1.2 Mb) [3]. Mimivirus has 979 protein-encoding sequences (CDSs), six tRNA genes and 33 non-coding RNA genes [4]. ii) Viruses that infect algae (phycodnaviruses) and have genomes up to ∼560 kb [5,6]. iii) Viruses, such as bacterophage G, that infect bacteria and have genomes up to ∼670 kb (∼498 kb is unique sequence) [7].

A recent report describes the newest girus, a lytic virus (named CroV) that infects the marine microzooplankton *Cafeteria roenbergensis* [1]. CroV has a ∼730 kb genome and contains 544 CDSs and 22 tRNAs encoding genes in the 618 kb central region of its genome. Other dsDNA-containing viruses with genomes larger than 300 kb are listed in Table 1. Viruses with genomes ranging from 100 to 280 kb, such as herpesviruses and baculoviruses, are not discussed in this commentary, and poxviruses, asfarviruses, iridoviruses, and ascoviruses are only briefly mentioned because of their evolutionary connection to some giruses. Another group of viruses with dsDNA genomes >500 kb are the polydnaviruses. However, these viruses are also not discussed because they lack some features typically associated with viruses, such as high gene density [8,9].

To place the size of these large viruses into perspective, the smallest free-living bacterium, *Mycoplasma genitalium*, encodes ∼470 CDSs [10]. Although estimates of the minimum genome size required to support life are ∼250 CDSs [11,12], some symbiotic bacteria such as *Carsonella ruddii* [13] and *Hodgkinia cicadicola* [14] have genomes of 160 kb and 144 kb, respectively. Thus, many large viruses have more CDSs than some single-celled organisms.

Except for bacteriophage G [15,16] and the chlorella virus *Paramecium bursaria* chlorella virus (PBCV-1) [17], giruses have only been discovered and characterized in the last few years. There are several reasons why large viruses were undetected. i) Classical virus isolation procedures include filtration through 0.2 μm pore filters to remove microorganisms, which also exclude many large viruses. ii) Large bacteriophages were missed by standard plaquing procedures because the high soft agar concentrations reduced virus diffusion and hence formation of visible plaques [18]. Although not true of the viruses discussed in this commentary, large viruses might also grow slower than smaller viruses and have lower burst sizes. Another issue is that many large viruses infect protists, and protists are only beginning to be examined for virus infections. Finally, the discovery of some large viruses was serendipitous; e.g., Mimivirus was initially believed to be a parasitic bacterium [3].

## 2. Many More Giruses Await Discovery

Metagenomic studies indicate that giruses are common in nature and wait to be discovered. For example, one study used the Sorcerer II Global Ocean metagenome to determine the frequency that DNA polymerase fragments could be assigned to virus groups. The results indicated that Mimiviruses were second in abundance to bacteriophages [19]. Another recent report identified 19 more giant viruses from diverse environments, including soil, that infect amoeba [20]. In yet another study using three proteins, other than DNA polymerase, as queries, phycodnaviruses were commonly found in the Sargasso Sea and the Global Ocean Survey metagenomes [21]. These results imply that giruses constitute a quantitatively important and ubiquitous component of marine DNA viruses. The newly characterized CroV virus that infects *Cafeteria roenbergensis* indicates that giruses infect protists besides amoeba and algae.

## 3. Role of Viruses in the Environment

In addition to the interesting biology associated with giruses that is briefly mentioned below, giruses along with smaller viruses play major roles in the ecology of aqueous environments, which is only now becoming apparent. Viruses are the most abundant biological entities on earth and are major drivers of nutrient and energy cycles on the planet (for detailed discussions see [22–24]). More than 50% of the CO_2_ fixed on the planet is by photosynthetic microorganisms, including cyanobacteria and microalgae (collectively referred to as phytoplankton). Current estimates are that at any one time ∼20% of phytoplankton cells are infected by viruses, including viruses that qualify as giruses. Additional components of this aquatic foodweb are microzooplankton that graze on these microorganisms, referred to as protistan grazers. CroV is the first virus to be characterized that infects a protistan grazer.

Two examples illustrate the importance of giruses in the phytoplankton community. The coccolithophore alga, *Emiliania huxleyi*, is one of the most abundant and widely distributed photosynthetic unicellular eukaryotes in the oceans. Coccolithophores produce skeletons of minute calcite platelets (called coccoliths); consequently, they are major contributors to the oceanic carbon cycle and thus to the flux of CO_2_ between the atmosphere and oceans. *E. huxleyi* cells periodically form huge blooms covering wide coastal and mid-oceanic areas at high latitudes in both the northern and southern hemispheres. Large viruses that infect *E. huxleyi* (named EhV viruses) are largely responsible for the termination of these blooms (e.g., [6]). This termination releases massive quantities of organic and inorganic matter to the water column, including detached coccoliths that ultimately settle to the ocean floor. One outcome of this ecological cycle is the White Cliffs of Dover in England.

The demise of *E. huxleyi* blooms also results in the release of dimethylsulfoniopropionate (DMSP) from the dying alga, which is cleaved by DMSP lyases; DMSP lyases are common in marine microorganisms. The cleavage products are acrylic acid and dimethylsulfide; dimethylsulfide is released into the atmosphere inducing cloud formation and rain. Thus, EhV infection of its host plays a significant role in climate conditions (e.g., [25]).

One interesting feature of EhV is that it only infects the diploid phase of the *E. huxleyi* life cycle. The haploid state is resistant to EhV [26].

## 4. Evolution of Giruses

Viruses classified in virus families *Mimiviridae*, *Phycodnaviridae*, *Poxviridae*, *Asfarviridae*, *Iridoviridae*, and *Ascoviridae* probably have a common evolutionary ancestor and are referred to as nucleocytoplasmic large DNA viruses (NCLDVs) [27–29]. Recently, another large virus named Marseillevirus (368 kb genome), which is distantly related to the iridoviruses and ascoviruses, was isolated from an amoeba and it will probably be assigned to a new NCLDV family [30]. The newly described CroV has been tentatively assigned to the *Mimiviridae* family because 32% or the CroVs are Mimivirus homologs [1].

Comparative analysis of 45 NCLDVs identified five common genes in all the viruses and 177 additional genes that are shared by at least two of these virus families [31]. The five common CDSs are the major capsid protein, a primase-helicase, a family B DNA polymerase, a DNA packaging ATPase and a transcription factor.

Although common ancestry of NCLDVs is generally accepted, there is disagreement on the size and morphology of its ancestor and how it evolved into the different virus families. Like cellular organisms, gene and genome duplication contributed to the large genome of Mimivirus and maybe other giruses [32]. A maximum-likelihood reconstruction of NCLDV evolution using 45 NCLDV genomes produced a set of 47 conserved genes, which were considered to be the minimum genome for the common ancestor [31]. NCLDVs were then proposed to evolve by losing some of these common genes and by acquiring new genes from their hosts and bacterial endosymbionts as well as by gene duplications. Another hypothesis proposes that the ancestral NCLDV was a huge virus or even a cellular organism that evolved primarily via genome contraction [33]. Finally, Filee *et al*. [34] proposed that NCLDVs evolved from a small DNA virus by acquiring genes from cellular sources.

There are also disagreements on the origin of the NCLDVs. For example, some researchers have suggested that NCLDVs should be considered the fourth kingdom of life [33,35], others have suggested that many NCLDV genes arose from the original gene pool that led to prokaryotes and eukaryotes [28], and still others have suggested that horizontal gene transfer has driven the evolution of their genomes [36]. One problem with this later suggestion is that probably only a small fraction of the viral genes came by gene transfer from cells. For example, ∼66% of the CDSs in the Mimivirus genome have no functional similarity to known proteins, suggesting that Mimivirus arose early in evolution. This phenomenon is not exclusive to Mimivirus, ∼60% of the ORFs of the large bacteriophage sk1 genome and 94% of the ORFs from white spot syndrome virus have no functional homologues to known proteins [37]. Also, some genes may have a viral origin and not a cellular origin [38]. In these instances, viruses may have contributed genes to the host, rather than the other way around.

Contributing to the discussion about NCLDV evolution is the discovery that the structure of the chlorella virus PBCV-1 major capsid protein (MCP) resembles MCPs from some smaller dsDNA viruses with hosts in all three domains of life, including human adenoviruses, bacteriophage PRD1, and a virus infecting an archaeon, *Sulfolobus solfataricus*. This structural similarity suggests that these three viruses might have an evolutionary connection to NCLDVs, despite the lack of amino acid sequence similarity among their MCPs (for a detailed discussion see [39,40]). If so, the formation of these viral lineages might have predated the divergence of Archaea, Bacteria and Eukarya.

Another interesting hypothesis is that a primitive NCLDV gave rise to the eukaryotic nucleus or *vice versa* [41,42]. Taken together, these hypotheses suggest that the NCLDVs, as well as other viruses, are ancient and have probably contributed significantly to the emergence and subsequent structure of modern cellular life forms [43].

Detailed reviews by Forterre [38] and Koonin and Yutin [29] on the evolution of large DNA viruses summarize much of the discussion on this subject.

## 5. Are Viruses Alive?

The discovery and characterization of giruses has revived a discussion of whether viruses should be considered to be living organisms, e.g., [44]. One perspective is the idea that one should actually compare the intracellular stage of viral replication (*i.e.*, the viral factory [45]), which is metabolically active, with cells, rather than the virion, which is metabolically inactive. Thus, Claverie has recommended that the “virus factory” be considered the actual organism when referring to a virus (for a detailed discussion see [2,46].

These ideas have stimulated a lively discussion as to whether the tree of life should include viruses (e.g., see [2,44,47], and Nat. Rev. Microbiol. (2009) 7, 14–27 [37] for seven commentaries on the subject).

## 6. Problems Associated with the Taxonomy of Giruses

The issue of the evolutionary origin of the NCLDVs also contributes to the difficulty of classifying some of these viruses into distinct families. Phylogenetic analysis of the DNA polymerase from four putative phycodnaviruses illustrates the problem. i) The DNA polymerase from three putative phycodnaviruses, CdV01, PpV01, and PoV01 (Table 1), is more similar to Mimivirus than to the other phycodnaviruses [19]. In fact, these authors suggest that Mimivirus relatives are probably large algal viruses. ii) The closest relative of the DNA polymerase from the fourth putative phycodnavirus, HcDNAV, is African swine fever virus [48]. However, if one conducts a similar analysis with the MCPs, the phylogenetic trees change [49]. Therefore, it is clear that these viruses, like the DNA bacteriophages [50], have been exchanging genes for eons. One potential venue for gene mixing is amoeba, which harbor many diverse microorganisms, including viruses; thus amoeba could serve as a “melting pot” for gene mixing, leading to new viruses, including large viruses with complex gene repertories of various origins [30,51].

Adding to the classification issue is the proposal that the taxonomic status of viruses should be elevated to the same level as cells by dividing the biological world into two classes of organisms, those encoding capsids, and those encoding ribosomes [44].

## 7. Diversity of Girus Lifestyles

**Figure 1:**
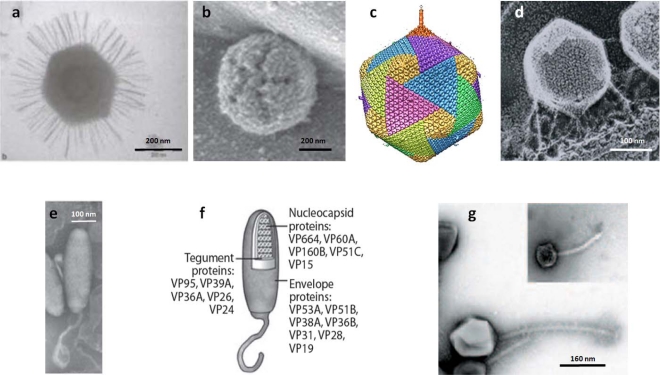
(**a**) Transmission electron micrograph of Mimivirus. (**b**) Atomic force microscopy of defibered Mimivirus. The unique star-faced vertex is clearly visible. (**c**) Five-fold averaged cryo-electron micrographs of virus PBCV-1 reveal a long, thin, cylindrical spike structure at one vertex and protrusions (fibers) extending from one unique capsomer per trisymmetron. (**d**) PBCV-1 attached to the cell wall as viewed by the quick-freeze, deep-etch procedure. Note fibers attach the virus to the wall. (**e, f**) Morphology of the *White spot shrimp virus* (WSSV) virion. (**e**) Negative contrast electron micrograph of intact WSSV virion with its tail-like extension. (**f**) Schematic based on panel *e* showing the layered structures of a WSSV virion, *i.e.*, envelope, tegument, and nucleocapsid. (**g**) Electron micrograph of bacteriophage G. The insert shows coliphage lambda to the same scale. Panel (**a**) is from [77], (**b**) is from [58], (**c**) is from [78], (**d**) is from [79], (**e**) and (**f**) are from [80], and (**g**) is from [50]. The figure is modified, with permission, from Figure 1 in the Annual Review of Microbiology, Volume 64, 83–99 [52].

One issue that is perhaps not appreciated by the virology community is that these large viruses, even members within the same family, can differ in morphology (see Figure 1) and lifestyle [52]. Examples of some lifestyle differences among the NCLDVs include: i) The infection process differs among the phycodnaviruses. Chlorella virus infection is bacteriophage-like. The viruses attach to a specific receptor on the chlorella wall at a unique vertex (Figure 1c) and digest the wall at the point of attachment. The internal virus membrane then presumably fuses with the host plasma membrane allowing the DNA and virion-associated proteins to be released to the inside of the cell. An empty capsid remains outside the cell [53,54].

In contrast, the entire particle of another phycodnavirus, EhV, which has an external membrane, enters the host intact via either endocytosis or an envelope fusion mechanism with the host plasma membrane and then rapidly disassembles [55]. In the case of Mimivirus, the entire particle is engulfed by the amoeba by phagocytosis. Once inside the phagosome, Mimivirus fuses with the lysosome. This lysosomal activity helps to open the viral capsid at a special vertex, called stargate (Figure 1b). The fusion of the particle’s internal membrane with the endocytic vacuole membrane forms a large membrane conduit through which the genome-containing Mimivirus core enters the cytoplasm.

ii) All NCLDVs are assembled in “virus factories” located in the cytoplasm. However, the role of the nucleus in the replication of NCLDVs varies. For example, Mimivirus, like the poxviruses, appears to carry out its entire life cycle in the cytoplasm [56]. The intracellular transcription site for the newly characterized CroV is unknown; however, CroV encodes eight putative DNA-dependent RNA polymerase II subunits and at least six transcription factors, suggesting that its replication may also be independent of the nucleus. In contrast, the nucleus probably plays an essential role in the replication of most of the phycodnaviruses and other NCLDVs. However, the nuclear role in virus replication may differ, even among the phycodnaviruses. For example, the chlorella viruses do not encode any CDSs resembling RNA polymerase subunits; in contrast, the algal virus EhV encodes six RNA polymerase subunits [57].

Mimivirus has provided another surprise for virologists; all known icosahedral viruses with a unique vertex package their DNA at the same vertex that releases DNA, e.g., tailed bacteriophage. However, Mimivirus is reported to package its DNA through a face-centered aperture rather than the vertex-centered stargate structure that is involved in DNA release [58]. If this result is verified, Mimivirus differs from other viruses with a unique vertex in which DNA exits and packages at the same portal.

iii) Exit of newly formed viruses from the cell can also differ among giruses. The phycodnavirus EhV buds from its host [55], whereas, intact and infectious PBCV-1 virions form inside the cell and nascent virions exit by lysis of the cell plasma membrane and cell wall [59].

Finally, it should be noted that most girus genomes do not integrate into their host genomes. The one exception is the phycodnavirus EsV, which infects the filamentous marine brown alga *Ectocapus siliculosus*; EsV has a lysogenic life cycle [60].

## 8. Girus Encoded Genes and Metabolic Pathways

Not surprisingly, giruses encode an amazing array of proteins and even metabolic pathways, as well as properties that are typically the function of the host. Of course, the function of more than 50% of girus CDSs are unknown. Because of space limitations, only a few unusual virus-encoded properties are mentioned to illustrate their diversity. i) Both CroV and Mimivirus, as well as other giruses, contain several CDSs involved in protein translation, including amino acyl tRNA synthases and translation initiation factors, as well as tRNAs [3,1]. ii) In contrast to other viruses that use the host machinery located in the endoplasmic reticulum and Golgi to glycosylate their glycoproteins, the chlorella viruses encode most, if not all, of the components to glycosylate their major capsid proteins. Furthermore, all experimental results indicate that chlorella virus glycosylation is independent of the endoplasmic reticulum and Golgi [61]. This property may also exist in other giruses because some of their CDSs are predicted to be glycosyltransferases.

Examples of unusual putative pathways encoded by giruses include: i) The newly described CroV genome has a 38 kb genomic fragment that encodes an entire biosynthetic pathway for 3-deoxy-D-manno-octulosonate (referred to as KDO) [1]. In Gram-negative bacteria, KDO is an essential core component of the lipopolysaccharide layer, linking lipid A to polysaccharides. The G + C content of this 38 kb fragment differs slightly from the remainder of the genome, which suggests it was acquired after the lineage split from the Mimivirus lineage. Other giruses encode enzymes involved in the synthesis of sugars, e.g., enzymes that synthesize fucose and rhamnose [62]. ii) The phycodnavirus EhV has seven CDSs that form a metabolic pathway that synthesizes spingolipids [63]. The EhV host, *Emiliania huxleyi*, also has genes encoding this pathway and obviously horizontal gene transfer occurred between EhV and *E. huxleyi* [63]. However, the direction of the transfer is unknown. The viral biosynthetic pathway is expressed during lytic infection and the resulting glycosphingolipids (GSLs) induce programmed cell death (PCD); PCD activates a host caspase-like activity that is required for EhV-86 replication. Susceptible hosts accumulate both algal and viral derived GSLs that may coordinate virus maturation, whereas resistant cells accumulate only algal derived GSLs. The viral GSLs accumulate in the viral envelope, and it is hypothesized that this mechanism activates virus release into the environment and subsequently induces PCD in surrounding algal cells that aids in termination of algal blooms [64]. This example of cell signaling by the *E. huxleyi*/EhV interaction suggests that aquatic viruses may control their environment in ways virologists and ecologists are only just beginning to fathom. iii) Some chlorella viruses encode three enzymes, including hyaluronan synthase, involved in the synthesis of the extracellular matrix polysaccharide hyaluronan; hyaluronan consists of alternating β1,4-glucuronic acid and β1,3-N-acetylglucosamine residues. Hyaluronan (also known as hyaluronic acid) accumulates on the external surface of the infected chlorella cells [65]. Previously hyaluronan had only been found in vertebrates and a few pathogenic bacteria. Other chlorella virus encoded CDSs are involved in chitin biosynthesis and chitin accumulates on the surface of cells infected with these viruses [66]. The function of these extracellular matrix polysaccharides is unknown. iv) The chlorella viruses also encode four proteins involved in polyamine biosynthesis [67].

Examples of some other unexpected proteins and enzymes encoded by giruses include: i) the chlorella viruses encode several ion channel and transporter proteins [68]. ii) The chlorella viruses encode many DNA methyltransferases and DNA restriction endonucleases [69]. iii) The newly described CroV encodes two photolyases. iv) Enzymes involved in various ubiquination functions are also common in many of these large viruses.

It should be emphasized that many of the virus encoded, unexpected CDSs mentioned above are still putative assignments because biochemical evidence is lacking. However, other girus encoded CDSs have been expressed and have the expected properties. Some chlorella virus encoded proteins are the smallest or among the smallest proteins of their family, e.g., a histone methyltransferase [70], an ornithine decarboxylase [71], a type II DNA topoisomerase [72], and a potassium ion channel protein [68,73]. Furthermore, phylogenetic analyses suggest some of these minimalist proteins might be evolutionarily precursors of more complex contemporary proteins. Despite their small sizes, the virus enzymes typically have many of the catalytic properties of larger enzymes. Their small size and the fact that they are often “laboratory friendly” have made them important models for mechanistic and structural studies (e.g., [74]).

## 9. Exploiting Girus Genes

The amino acid differences between girus orthologs, which probably results from the long evolutionary history of these viruses, can be exploited to aid in understanding protein function. The following example illustrates this property. Many electrophysiological experiments have been conducted in *Xenopus* oocytes on the 94 amino acid chlorella virus PBCV-1 encoded potassium ion channel protein Kcv (reviewed in [68]). Kcv-like genes were cloned and sequenced from 40 additional viruses that infect the same host; 16 amino acid substitutions occurred among the 94 amino acids, producing six new Kcv-like proteins that formed functional potassium ion selective channels in *Xenopus* oocytes. However, the biophysical properties of some of these Kcv channels differed from PBCV-1 Kcv, including altered current kinetics with K^+^ and Rb^+^ and altered sensitivity to ion channel blockers. The amino acid differences, together with the altered electrophysiological properties, served to guide site-directed amino acid substitutions, either singularly or in combinations, to identify key residues that conferred specific properties to Kcv. Other chlorella virus encoded gene products await similar exploitation.

## 10. Concluding Comments

Although giruses are probably ancient, they are relatively new to virologists. Even with our limited knowledge, research efforts on large viruses are contributing scientific and economic benefits. For example, chlorella viruses, which encode as many as 400 CDSs, are sources of new and surprising proteins, including commercially important enzymes such as DNA restriction endonucleases [69]. The chlorella viruses are also a source of genetic elements for genetically engineering other organisms. Examples include i) promoter elements that function well in both monocots and dicots of higher plants, as well as bacteria [75]; and ii) a translational enhancer element from a chlorella virus that functions well in *Arabidopsis* [76].

The sequence of some girus host genomes have either recently been completed or are in the process. Annotation of these host sequences will contribute to studies on giruses. However, a major obstacle to studying these viruses is that currently none of them can be genetically modified by molecular techniques. The development of successful and reproducible host transformation procedures should lead to the molecular genetic analysis of these viruses, which would lead to major advances in the understanding of these fascinating viruses.

## Figures and Tables

**Table 1:** Giruses and some of their properties

**Classification**		**Genome**						**Virion**			**Host**

**Virus family**	**Type member**	**Size (kb)**	**G+C%**	**% coding**	**Shape**	**Predicted CDS**	**tRNA genes**	**Nucleocapsid symmetry**	**Nucleocapsid diameter (nm)**	**Lipids**	**Kingdom**

Mimiviridae	*Acanthamoeba polyphaga* mimivirus (APMV)	1181	27	86	circular	911	1	isometric	500	Yes	Protozoa
	Mamavirus	∼1200	28	N/A	circular	N/A	N/A	isometric	N/A	Yes	Protozoa
	*Cafeteria roenbergensis* virus (CroV)	730	23	90	N/A	544	22	isometric	∼300	N/A	Chromista
Myoviridae	*Pseudomonas chlororaphis* phage 201φ2-1	317	45	93	Linear	461	1	isometric	122	None	Proteobacteria
	*Bacillus megaterium* phage G	670	N/A	N/A	N/A	N/A	N/A	isometric	N/A	N/A	Firmicutes
Nimaviridae	White spot syndrome virus 1 (WSSV1)	305	41	92	circular	531	0	helical	N/A	Yes	Animalia
Phycodnaviridae	*Chlorovirus* PBCV-1	331	39	90	linear	366	11	isometric	190	Yes(I)	Plantae
	*Chlorovirus* NY2A	369	40	92	linear	404	7	isometric	N/A	Yes(I)	Plantae
	*Chlorovirus* AR158	345	40	92	linear	360	6	isometric	N/A	Yes(I)	Plantae
	*Chlorovirus* FR483	321	44	93	linear	335	9	isometric	N/A	Yes(I)	Plantae
	*Chlorovirus* MT325	321	45	N/A	Linear	331	10	isometric	N/A	Yes(I)	Plantae
	*Ectocarpus siliculosus virus 1 (EsV-1)*	336	51	70	circular	240	0	isometric	N/A	N/A	Plantae
	*Emiliania huxleyi virus 86 (EhV86)*	407	40	90	circular	472	6	isometric	N/A	N/A	Chromista
	*Chrysochromulina ericina virus 1 (CeV01)	∼510	N/A	N/A	N/A	N/A	N/A	isometric	160	N/A	Chromista
	*Pyramimonas orientalis virus 1 (PoV-01)	∼560	N/A	N/A	N/A	N/A	N/A	isometric	222x180	N/A	Plantae
	*Phaeocystis globosa virus 1 (PgV group 1)	∼466	40–52	N/A	linear	N/A	N/A	isometric	150–190	Yes(I)	Chromista
	*Phaeocystis pouchetii virus 01 (PpV-01)	∼485	N/A	N/A	N/A	N/A	N/A	isometric	220	Yes(I)	Chromista
	*Heterocapsa circularisquama viru (HcDNAV)	356	N/A	N/A	N/A	N/A	N/A	isometric	197	N/A	Protozoa
	*Ectocarpus fasciculatus virus a* (EfasV)	∼340	N/A	N/A	N/A	N/A	N/A	isometric	N/A	N/A	Chromista
	*Myriotrichia clavaeformis virus a* (MclaV)	∼340	N/A	N/A	N/A	N/A	N/A	isometric	N/A	N/A	Chromista
Poxviridae	Canarypox virus (CNPV)	∼360	30	90	linear	328	0	isometric	160–190	Yes	Animalia
N/A	Marseillevirus	368	45	89	circular	457	N/A	isometric	250	N/A	Protozoa

The genome sizes with ∼ in front of the number have not been completely sequenced and annotated.

* These viruses are currently listed in the Phycodnaviridae because they infect algae. However, they may be moved into another family (e.g., see reference [29]).

Yes(I) = Yes, lipids are found with the virion and internal to the nucleocapsid.

This table is modified, with permission, from Table 1 in the Annual Review of Microbiology, Volume 64, 83–99 [52].
